# Horner Syndrome Secondary to Thyroid Surgery

**DOI:** 10.1155/2017/1689039

**Published:** 2017-01-04

**Authors:** Meliha Demiral, Ciğdem Binay, Enver Simsek, Hüseyin Ilhan

**Affiliations:** ^1^Department of Pediatric Endocrinology, School of Medicine, Eskişehir Osmangazi University, Eskişehir, Turkey; ^2^Department of Pediatric Surgery, School of Medicine, Eskişehir Osmangazi University, Eskişehir, Turkey

## Abstract

Horner syndrome (HS), caused by an interruption in the oculosympathetic pathway, is characterised by myosis, ipsilateral blepharoptosis, enophthalmos, facial anhydrosis, and vascular dilation of the lateral part of the face. HS is a rare complication of thyroidectomy. A 15-year-old female patient presented with solitary solid and large nodule in the right thyroid lobe. Ultrasound-guided fine-needle aspiration was performed and the cytological examination results were undefined. The patient underwent a total thyroidectomy. On postoperative day 2, she developed right-sided myosis and upper eyelid ptosis. HS was diagnosed. Interestingly, the patient exhibited an incomplete clinical syndrome with the absence of vasomotor symptoms. We herein report a case of HS in a 15-year-old female patient after total thyroidectomy. The possible causes of HS were ischaemia-induced nerve damage and stretching of the cervical sympathetic chain by the retractor during thyroidectomy. Clinicians should be aware of the possibility of this rare but important surgical complication.

## 1. Introduction

Horner syndrome (HS) was first described in 1869 by Johann Friedrich Horner [1]. The typical clinical features include myosis, ipsilateral blepharoptosis, enophthalmos, facial anhydrosis, and vascular dilation of the lateral part of the face. These conditions result from an interruption in the oculosympathetic pathway. The present literature review reveals that compression of the cervical sympathetic chain by large benign or malignant goitres may present as HS, and this has mostly been reported in adult patients [2–4]. However, HS has rarely been reported as a complication of thyroid surgery [5, 6]. We herein report a case of HS following total thyroidectomy in a 15-year-old girl.

## 2. Case Report

A 15-year-old female patient presented to the Pediatric Endocrinology Division at the Medical Faculty Hospital of Eskisehir Osmangazi University with a 2-month history of a noticeable neck mass. Physical examination revealed an enlarged thyroid gland with a palpable nodule in the right thyroid lobe. The patient had no other complaints related to thyroid disease. Her medical history was unremarkable. She had no history of previous radiation in her cervical region and no family history of thyroid disease. On physical examination, her weight was 64 kg (+1.16 standard deviation score), her height was 155 cm (−1.15 standard deviation score), her body mass index was 26.6 kg/m^2^, and her blood pressure was 110/80 mmHg. A palpable nontender nodule measuring 35 mm × 20 mm completely covered the right thyroid lobe region. There was no evidence of cervical lymphadenopathy.

Thyroid function tests revealed thyroid stimulating hormone and free thyroxin levels of 1.0 mcIU/mL and 1.2 ng/dL, respectively. Thyroid peroxidase antibody and thyroglobulin antibody titres were negative. Thyroid ultrasonography showed markedly enlarged right and left lobes measuring 33 × 21 × 50 mm and 14 × 11 × 41 mm, respectively. A 31 × 19 × 38 mm solid nodule completely covered the right lobe with a hypoechoic halo. The patient underwent ultrasound-guided fine-needle aspiration, and the cytological examination results were undefined.

**Figure 1:**
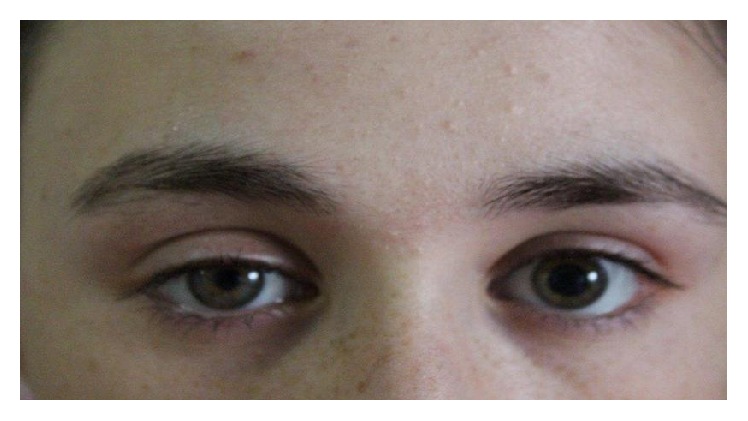
Myosis and eyelid ptosis were noted on the right side.

The patient then underwent a total thyroidectomy. On postoperative day 2, she developed right-sided myosis and upper eyelid ptosis (Figure 1). However, facial anhydrosis, enophthalmos, and a bitonal voice were not detected. Her extraocular eye movements and visual acuity were normal. Brain magnetic resonance imaging, chest X-rays, neck computed tomography, and single-fibre electromyography showed no distinct pathology. No other complications such as bleeding, wound infection, vocal cord palsy, or findings of hypoparathyroidism were detected. Histological examination of the tissue obtained from the thyroidectomy revealed nodular thyroid hyperplasia. Euthyroidism was achieved by L-thyroxin replacement at a dose of 150 mcg/day. The patient has been followed up for 8 months. Six months after the thyroid surgery, physical examination findings were normal, and the patient exhibited no myosis or ptosis.

## 3. Discussion

The aetiologies of HS in childhood are classically divided into congenital and acquired causes. The major cause of acquired HS is postsurgical complications of the neck and thorax [7]. Noniatrogenic cases can be attributed to infection, trauma, vascular anomalies, and neoplasms such as sympathetic paraganglioma, neuroblastoma, schwannoma, and Ewing sarcoma [8–11].

Horner syndrome is a rare complication of thyroid disease. The majority of aetiologies involve compression of the cervical plexus. Thyroid carcinoma accounts for 21% of cases [12]. Of the reported cases, multinodular goitre, Riedel's and Hashimoto's thyroiditis, thyroid adenoma, and thyroid lymphoma have been associated with HS. Most of the reported cases occurred in an adult population [12, 13].

Horner syndrome following thyroid surgery is an extremely rare complication with an incidence of less than 0.2% to 0.3% among patients undergoing thyroidectomy [3]. Smith and Murley [14] reported 25 cases involving patients with iatrogenic injures following either partial or total thyroidectomy. Buhr et al. [15] also reported three cases of HS following modified radical neck dissection for medullary thyroid cancer. It has been postulated that HS might be caused not only by direct mechanical stress or compression of the stellate ganglion but also indirectly through anastomosis of the recurrent nerve, laryngeal superior nerve, and sympathetic nervous branches around the inferior thyroid artery [6]. The possible causes of HS after thyroid surgery have been attributed to the postoperative formation of a hematoma compressing the cervical sympathetic chain, ischaemia-induced damage caused by a lateral ligature on the inferior thyroid artery trunk, stretching of the cervical sympathetic chain by the tip of the retractor, and damage to communication between the cervical sympathetic chain and the recurrent laryngeal nerve during its identification [6].

In most cases, the HS is incomplete, exhibiting the absence of vasomotor symptoms, as in our case. The present case had some characteristics similar to those of previous cases, such as the onset of HS on postoperative day 2 and the lack of symptoms related to vascular or sweating dysfunction [16, 17].

The prognosis of HS has been proposed to be poor depending on the particular mechanism of injury. However, if the injury is related to hematoma formation, inflammation, or peripheral ligature of the inferior thyroid artery branches, the HS may spontaneously resolve. In the present case, postoperative hematoma and adherence were ruled out by neck ultrasonography and computed tomography. The nonrecurrent laryngeal nerve, which is rarely observed during thyroidectomy, is at high risk for damage [18]. The best way to avoid morbidity is routine identification of this nerve. About 70% of patients who develop HS after thyroidectomy have permanent damage or incomplete recovery, and the remaining 30% recover completely, but only after a much longer time (20 days to 15 months) [6]. The HS in our patient was reversible, and all findings of HS completely disappeared.

In conclusion, we have reported a case with HS resulting from thyroid surgery for benign pathology. While HS appears to be a very rare complication of thyroid surgery, clinicians should be aware of the possibility of this rare but important complication.
